# Effects of different combinations of pre‐ and post‐grazing heights on herbage mass and nutrient reserves of *Leymus chinensis* in Northeast China

**DOI:** 10.1002/ece3.11336

**Published:** 2024-05-06

**Authors:** Chengzhen Zhao, Xiao Chang, Qiang Li, Rongzhen Zhong, Daowei Zhou

**Affiliations:** ^1^ Jilin Province Feed Processing and Ruminant Precision Breeding Cross regional Cooperation Technology Innovation Center Jilin Provincial Laboratory of Grassland Farming State Key Laboratory of Black Soils Conservation and Utilization Northeast Institute of Geography and Agroecology, Chinese Academy of Sciences Changchun China; ^2^ University of Chinese Academy of Sciences Beijing China

**Keywords:** apical meristem, carbohydrates reserves, defoliation height, herbage mass, *Leymus chinensis*

## Abstract

The preservation or removal of apical meristem in *Leymus chinensis* is contingent upon grazing intensity and has a significant impact on above‐ and belowground biomass, nutritive value, and sustainability of *L*. *chinensis* grassland. However, this topic remains understudied. Therefore, a manipulative trial was conducted to induce grazing defoliation through mowing, where two post‐grazing heights (preservation or removal of the apical meristem) and four pre‐grazing plant heights (i.e., 18, 24, 31, and 35 cm) are combined factorially to create gradients of grazing intensities, resulting in a total of eight treatments. Additionally, two identical control treatments are also incorporated. Our results showed that apical meristem removal at various pre‐grazing heights resulted in varying degrees of increased grazing intensities, thereby enhancing the nutritive value of *L*. *chinensis*. However, this practice also led to detrimental effects on the plant's carbohydrates reserve as well as herbage mass. The results indicated that although defoliation in treatments involving apical meristem removal resulted in the highest number of frequent cuttings, it did not confer any advantages in terms of herbage mass and nutrient preserves, except for herbage nutritive values when compared to treatments involving apical meristem preservation. The apical meristem preservation treatments demonstrated the highest CP yield over a 2‐year period compared to the apical meristem removal treatments. Furthermore, within these apical meristem preservation treatments, only when the pre‐grazing height is 35 cm and post‐grazing height is 17 cm, there is no significant decrease in above‐ and belowground biomass. This establishes this specific defoliation regime as an optimal and effective management strategy for *L*. *chinensis* grassland.

## Graphical abstract

The apical meristem plays a crucial role in the growth of *L*. *chinensis*, as its removal leads to death and reproduction through tillers, while its preservation ensures survival and continued growth via regenerative properties. Our findings suggest that preserving the apical meristem of *L*. *chinensis* leads to increased herbage mass and greater persistence of *L*. *chinensis* grassland, in contrast to removing it.

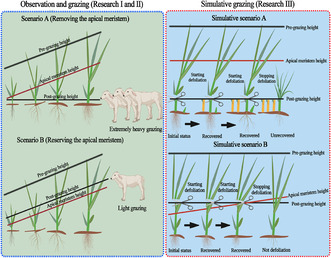

## 1 INTRODUCTION

The typical grassland, which is dominated by *Leymus chinensis* (Trin.) Tzvel, covers the majority of the eastern Eurasian temperate grassland at the northern boundary of China (Wang et al., 2017), which plays an important role in animal husbandry production and ecological security (Jiang & Wang, 2022; Pan et al., 2018). *L*. *chinensis* accounts for about 90% of the total aboveground DM yield with high acceptance and forage value in this region; moreover, it is highly tolerant of the region's arid conditions (Ren et al., 2014). Therefore, from the economic and ecological attributes of *L*. *chinensis*, this grass has been of keen interest to ecologists and agronomists for the past several decades. However, there is still lack of detailed information on natural grassland use to take into account its yield, nutritive value, and sustainability.

Traditionally, both livestock grazing and haymaking are the primary economic use of natural pastures in arid and semi‐arid natural grasslands in Northeast China, of which grazing is considered the most economic use of natural grassland (Haynes et al., 2014; Nave et al., 2014). Different grassland utilization methods can lead to different objectives. In the temperate grasslands of the northern hemisphere, the annual autumn hay‐harvesting practice involves mowing in mid‐August with a stubble height of 6 cm, resulting in highest yield of herbage dry matter (DM), albeit at a comparatively lower nutritive value (Zhao et al., 2021). Moreover, the study conducted by Yang et al. (2020) involved defoliation treatments with six different stubble heights: 14, 12, 10, 8, 6 cm, and less than 0.3 cm above the ground surface. These treatments were implemented once a year during the peak plant biomass period (mid‐August) from 2013 to 2016. The results revealed that after 3 consecutive years of defoliation, the treatment with a defoliation height of 14 cm significantly enhanced forage yield in the subsequent 4th year compared to treatments with a defoliation height of only 0.3 cm. Therefore, increasing defoliation intensity can significantly enhance the nutritive value of the forage, but it ultimately results in a reduction in DM yield in the long term (Martins et al., 2021). This is attributed to the substantial removal of aboveground photosynthetic tissues resulting from high defoliation intensity (Kleijn et al., 2005; Yao et al., 2019). Summarily, considering only one parameter alone (i.e., forage yield, forage nutritive value, and grassland persistence) might not obtain the optimal grazing scheme. In case herbage yield and its nutritive value are opposite in response to different defoliation intensities, it may be possible to identify an optimal defoliation intensity that increases the herbage mass (CP yield) and enhances the sustainability of *L*. *chinensis* grassland.

The grazing intensity plays a crucial role in effective grazing management, as there is typically an inverse relationship between defoliation intensity and both the forage yield and nutritive value of perennial grasses. Previous studies have predominantly employed mechanical methods to determine grazing or cutting intensity and examined its impact on the entire grassland ecosystem, neglecting to consider its effect on individual *L*. *chinensis* plants, particularly with regard to variations in their apical meristem. The apical meristem of *L*. *chinensis* plays a crucial role in its survival and growth, and the preservation or removal of the apical meristem determines the forage yield and nutritive value. Removal of the apical meristem leads to the demise of individual *L*. *chinensis* plants and stimulates rhizome production of more young tillers (Liu et al., 2019; Yang et al., 2020). In this case, grassland primarily consists of young tillers, thereby increasing forage nutritive value. Conversely, when the apical meristem remains intact, *L*. *chinensis* thrives (Liu et al., 2019), resulting in older plants dominating the grassland and negatively impacting forage nutritive value but benefiting plant nutrient reserves. During the grazing scenario, whether the apical meristem of *L*. *chinensis* will be consumed by animals is contingent upon grazing intensity. Previous research has demonstrated that sheep exhibit selective foraging behavior, preferring young leaves over stems or older foliage (Szymczak et al., 2020), thereby potentially preserving the apical meristem situated at the stem apex. However, previous studies have also indicated that with increasing grazing intensity, particularly in cases of overgrazing, there is an elevated likelihood of re‐grazing on stubble from *L*. *chinensis* (Liu et al., 2004), which may result in removal of its apical meristem.

The preservation or removal of the apical meristem also influences nutrient mobilization. Following the preservation of the apical meristem, there is an immediate mobilization of soluble sugars in storage organs, particularly stubbles after grazing, leading to a reallocation of resources toward younger and more efficient leaves rather than older leaves or stems. This reallocation is favorable for herbage regrowth (Zhao et al., 2009). However, once the apical meristem is removed due to high grazing intensity, the plant relies solely on nutrient reserves in the rhizomes for supporting new tiller growth. Over time, these nutrient reserves in the rhizomes will become depleted (Mikola et al., 2009; Wiley et al., 2019). Although the preservation and removal of the apical meristem of *L*. *chinensis* was common phenomenon in grazing or mowing practice in meadow or typical grassland, hardly any study compared the response of plants to preserving and removing the apical meristem of *L*. *chinensis*, which has limited our ability to find the best grazing regime to maintain sustainable grassland management.

In present study, we simulated a grazing experiment through mowing, encompassing various combinations of pre‐grazing plant heights (spanning from poorly developed to well‐developed stages) and post‐grazing plant heights (involving both preservation and removal of apical meristem in *L*. *chinensis* to establish gradients of defoliation intensities). We select typical rhizome‐type grass, *L*. *chinensis*, as a model to investigate the impacts of defoliation height, that of preservation and removal of the apical meristem, on herbage mass and nutrient storage in *L*. *chinensis* grassland. Specifically, we addressed the following two main research questions: (1) How does the preserving versus removing apical meristem affect CP and fiber yield, as well as soluble sugar and starch reserves of *L*. *chinensis* and their relationship? (2)What is the optimal grazing management strategy for *L*. *chinensis* grassland to ensure annual sustainability? We hypothesize that the removal treatments of the apical meristem of *L*. *chinensis* may result in higher CP yield in the 1st year compared to the preservation treatments of apical meristem. However, in the 2nd year, there is a noticeable decrease in CP yield for the removal treatments of apical meristem, leading to a significant reduction compared to the preservation treatments.

## 2 MATERIALS AND METHODS

### 2.1 Site description

The study site is located in the Changling Grassland Ecosystem Research Station at Songnen Plain, NE, China (44°34′ N, 123°35′ E; 145 m above sea level). This area is characterized by a temperate, semi‐arid continental monsoon climate. The annual mean daily temperature is 5.9°C, with the annual mean maximum and minimum daily temperatures reaching 6.4 and 4.6°C, respectively. The annual average precipitation is 427 mm, with 70%–80% of total precipitation occurring between June and September. The annual pan evaporation approximates 1600 mm. The frost‐free period is approximately 150 days (April–September). The soil type was alkali saline, mainly characterized as a Salic Solonetz in the World Reference Base for Soil Resources. The pH of the soil ranges from 8.0 to 11.0. The focus of this study was on *L*. *chinensis*, a native C_3_ perennial rhizomatous grazing grass that dominates in Northeast China, with a total distribution covering 1.67 × 10^7^ hm^2^. The study area is characterized by meadow grassland, where *L*. *chinensis* thrives with a height ranging from 40 to 60 cm. In this area, its aboveground dry matter yield during the peak season reaches 200–250 g/m^2^, accounting for over 95% of the total aboveground biomass. In addition to *L*. *chinensis*, the dominant plant associations include *Phragmites communis*, *Chloris virgata*, *Suaeda salsa*, and *Puccinellia tenuiflora*. Prior to commencement of the study, the traditional grassland management practices are grazing and autumn hay making for the past decades.

### 2.2 Experimental design and treatments

A manipulative trial was conducted to stimulate grazing defoliation through mowing, where the post‐grazing heights/defoliation heights (reservation or removal of the apical meristem) and pre‐grazing plant heights are combined factorially. The pre‐cutting heights were 18, 24, 31, and 35 cm, while their corresponding apical meristem heights of 1.7, 5.8, 9.5, and 13.7 cm were determined through meticulous observation of plant growth that effectively encompassed the entire spectrum of plant development. The post‐grazing heights (6, 10, 13.5, and 17 cm) for the preservation treatments of apical meristems were determined by grazing sheep to ensure that no apical meristems were removed at each corresponding pre‐grazing height. Additionally, a consistent post‐grazing height of 2 cm was artificially set to effectively remove the apical meristem at each pre‐grazing height. A more detailed description for determining the pre‐ and post‐grazing height is provided in Supporting Information.

**FIGURE 1:**
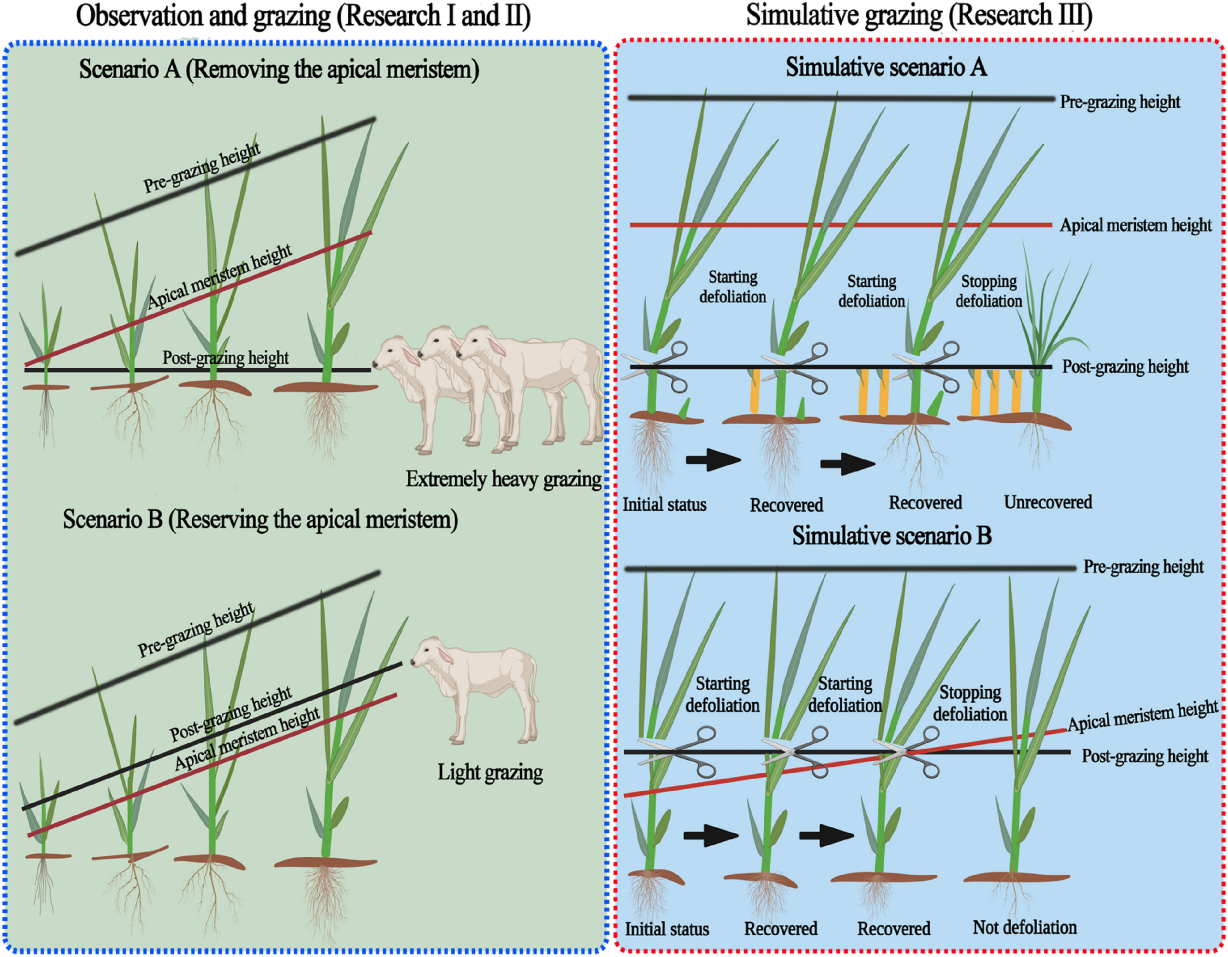
The graphical abstract of the entire experiment.

The treatments in the simulative grazing experiment involve manipulating combinations of pre‐ and post‐grazing plant heights to establish gradients of defoliation intensities. The treatments are described in detail as follows: the four preservation treatments for the apical meristem contain (1) the combination of a post‐grazing height of 6 cm and a pre‐grazing height of 18 cm; (2) the combination of a post‐grazing height of 10 cm and a pre‐grazing height of 24 cm; (3) the combination of a post‐grazing height of 13.5 cm and a pre‐grazing height of 31 cm; and (4) the combination of a post‐grazing height of 17 cm and a pre‐grazing height of 35 cm. Another four removal treatments for the apical meristem contains (5) the combination of a post‐grazing height of 2 cm and a pre‐grazing height of 18 cm; (6) the combination of a post‐grazing height of 2 cm and a pre‐grazing height of 24 cm; (7) the combination of a post‐grazing height of 2 cm and a pre‐grazing height of 31 cm; and (8) the combination of a post‐grazing height of 2 cm and a pre‐grazing height of 35 cm. Additionally, the two identical control treatments were also included, involving a single mowing in mid‐August following the traditional annual autumn hay‐making practice of Northeast China. The study was randomized block design and four replicate blocks were established in an area with fairly uniform vegetation and flat terrain in 2020, and each block includes ten 4 m × 4 m plots, separated by 1 m aisles, making 40 plots in total, representing 10 treatments, each replicated four times. The graphical abstract depicting the entire experiment is presented in Figure 1.

To provide a concise overview of the defoliation principles involving mowing dates, as well as regrowth days and number of mowing each treatment, we take treatment (the combination of a post‐grazing height of 6 cm and a pre‐grazing height of 18 cm) and treatment (the combination of a post‐grazing height of 2 cm and a pre‐grazing height of 18 cm) that underwent defoliation in 2020 as examples. The treatment (the combination of a post‐grazing height of 6 cm and a pre‐grazing height of 18 cm) in 2020 involved four mowings: the first on May 15, 2020, when the pre‐grazing height of *L*. *chinensis* reached 18 cm (with an apical meristem height of 1.7 cm); the second on June 4, 2020, when the pre‐grazing height recovered to 18 cm (with an apical meristem height of 2.7 cm); the third on June 27, 2020, when the pre‐grazing height recovered to 18 cm (with an apical meristem height of 4.0 cm); and finally, the fourth on July 15, 2020, when the pre‐grazing height recovered to 18 cm (with an apical meristem height of 4.5 cm). Therefore, this treatment, a post‐grazing height of 6 cm was consistently maintained to ensure preservation of the plant's apical meristem during each mowing operation. Additionally, the herbage in this treatment was harvested on September 30, 2020, at a defoliation height of 6 cm, aligning with the traditional practice employed by herdsmen during autumn hay harvesting. Therefore, mowing numbers for this treatment were five in the growing season in 2020 alone. The treatment (the combination of a post‐grazing height of 2 cm and a pre‐grazing height of 18 cm) in 2020 involved four mowings: the first on May 15, 2020, when the pre‐grazing height of *L*. *chinensis* reached 18 cm; the second on June 4, 2020, when the pre‐grazing height recovered to 18 cm; the third on June 30, 2020, when the pre‐grazing height recovered to 18 cm; and the fourth on August 19, 2020, when the pre‐grazing height recovered to 18 cm. Therefore, in this treatment, a post‐grazing height of 2 cm was sufficient to ensure removal of the plant's apical meristem during each mowing operation, no matter of the apical meristem height was. Additionally, the herbage in this treatment was harvested on September 30, 2020, at a defoliation height of 6 cm, aligning with the traditional practice employed by herdsmen during autumn hay harvesting. Therefore, mowing number for this treatment was also five in 2020 alone.

The dates of plant sampling and mowing events for all treatments across both years are summarized in Table S1. The pre‐grazing height and apical meristem height of all treatments at each mowing date across both years were documented in Table S2.

### 2.3 Field sampling and measurements

In 2020 and 2021, just before each mowing date, three 1 m × 1 m quadrats were randomly placed within each plot and all plant materials were mowed manually using scissors. All plant materials from each quadrat were bulked into one sample. *L*. *chinensis*, which was brought to the laboratory, and over‐dried at 65°C for 72 h and weighed. Three samples in each plot averaged as the DM yield per plot. Then, *L*. *chinensis* from each plot were combined and ground to pass through a 1‐mm screen, and ground samples were stored at 4°C prior to determining crude protein (CP), neutral detergent fiber (NDF), acid detergent fiber (ADF), and acid detergent lignin (ADL). The cumulative DM yield of *L*. *chinensis* of each treatment was calculated as the total DM yield across all mowing dates within each year. The nutritive value of *L*. *chinensis* of each treatment was averaged across all mowing dates within each year.

In 2020 and 2021, just before each mowing date (except for September 30), 10 consistent individual tillers of *L*. *chinensis* were randomly selected in each plot and clipped at ground surface. Each individual tiller was separated into leaves and stems (stem and sheath) samples in separate paper bags. All samples were dried at 65°C for 72 h and weighed. The biomass of 10 leaves and stems of individual tillers from each plot were averaged as leaf and stem biomass per plot, respectively. Then, the leaves and stems of the *L*. *chinensis* individuals from each plot were combined, pulverized, and passed through a 40‐mesh sieve for soluble sugar and starch analysis. All involved parameters of each treatment averaged cross‐mowing dates within each year.

*Leymus chinensis* root samples were collected on September 30, 2021. Three sods from each plot were meticulously excavated using an 8‐cm‐diameter core sampler to obtain root system at a depth of 0–30 cm. Subsequently, extraneous roots and soil adhered to the roots were carefully eliminated. The root samples underwent thorough washing with distilled water to eliminate dust and were dried at 65°C for 72 h before being weighed. The belowground biomass per plot was determined by averaging the biomass of three root samples from each plot.

### 2.4 Plant chemical analysis

Total nitrogen (N) content was determined using the Kjeldhal method as described by Jones (1991), and total N was multiplied by a factor of 6.25 to estimate CP content. NDF and ADF contents were determined using the method described by Van Soest et al. (1991). The content of acid detergent lignin (ADL) in the residues, which were obtained after measuring NDF and ADF, was determined by treating with 72% sulfuric acid at room temperature for 3 h using a Daisy Incubator (Ankom Technology Corp.). The CP yield was obtained by multiplying the corresponding DM yield with the CP content, and the cumulative CP yield of each treatment was determined by summing up the DM yield of all mowing dates within each year. The method for calculating cumulative yields of ADF, NDF, and ADL is identical to that used for cumulative CP yield. The soluble sugar and starch contents were determined using Comin Biochemical Test Kits (Comin Biotechnology Co., Ltd., Suzhou, China) according to the manufacturer's instructions (Liang et al., 2019). After extracting three times for each sample, the supernatant was used for soluble sugar determination and the residue was used for starch measurement. The accumulation of soluble sugars or starch in each component (leaves or stems) was calculated by multiplying the content of each with the corresponding biomass. The final contents and accumulations of leaf or stem soluble sugar and starch of each treatment were determined by averaging all mowing dates within each year.

### 2.5 Statistical analyses

As explanatory variables we used: (i) pre‐grazing plant height; (ii) post grazing plant heights; and (iii) year. A linear mixed‐effects model (LMM) was employed to analyze the effects of explanatory variables on all variables related to above‐ and belowground biomass, herbage mass, herbage quality, and herbage nutrient reserves. The fixed effects included pre‐ and post‐grazing plants height, while the random effect accounted for experimental block. Additionally, repeated measures were considered over multiple years within the same experimental plot. Furthermore, assessments were conducted to evaluate normality as well as heterogeneity/homogeneity of variance. The regression analysis employed in this study utilized the pre‐grazing treatments as the independent variable (*x*‐axis), while the observed variables were represented by two separate curves of post‐grazing treatments on the dependent variable (*y*‐axis). In order to present the y‐axis values in a regression analysis, the observed variables of both post‐grazing treatments were averaged across all cutting frequencies within each year and further averaged across 2 years. The selection of regression models was based on minimizing the AIC. The statistical significance of treatment differences was determined using a *p*‐value threshold of ≤.05. All statistical analyses were performed using SPSS16.0, while all figures were generated with Origin 2023 Pro and Bio‐render.

## 3 RESULTS

### 3.1 Above‐ and belowground biomass of *Leymus chinensis*

**FIGURE 2:**
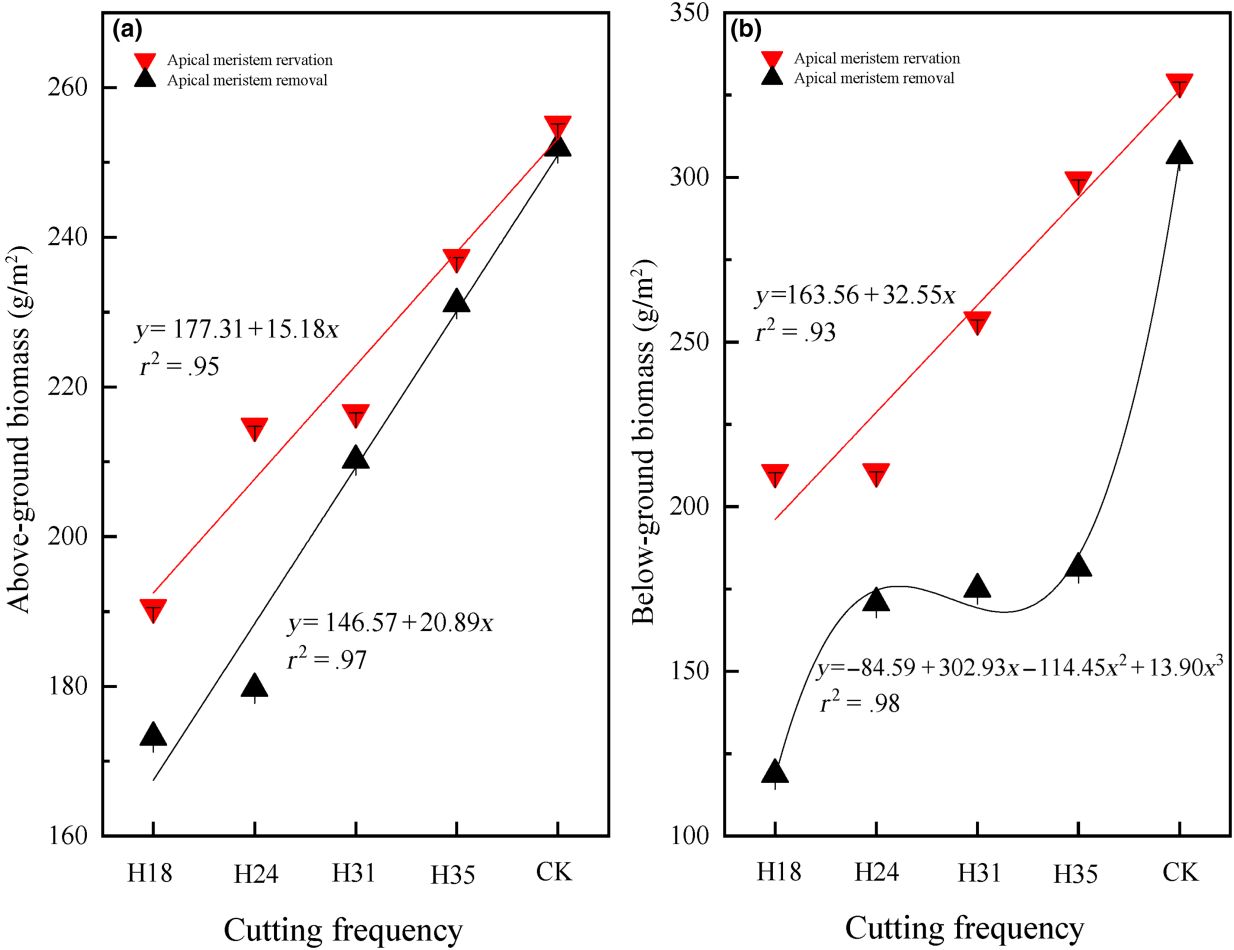
The above‐ (a) and belowground (b) biomass of *Leymus chinensis* in response to different pre‐grazing heights under two post‐grazing heights (apical meristem reservation and removal). The presented data in the figure represent the average values for each combination of pre‐ and post‐grazing height, considering all cutting frequencies within each year, and further averaged across 2 years. *Note*: H18 represents a pre‐grazing height of 18 cm; H24 represents a pre‐grazing height of 24 cm; H31 represents a pre‐grazing height of 31 cm; H35 represents a pre‐grazing height of 35 cm; CK represents no one cutting in mid‐August as common practice; Apical meristem reservation denotes the post‐grazing height reserved for the apical meristem; Apical meristem removal denotes the post‐grazing height removed for the apical meristem.

Both pre‐grazing height and post‐grazing height exert a significant influence on above‐ and belowground biomass (Table 1). Irrespective of the preservation or removal of the apical meristem in *L*. *chinensis*, there is a linear increase in aboveground biomass with an elevation in pre‐grazing height (Figure 2a). The belowground biomass for apical meristem conservation exhibits a linear increment as pre‐grazing height rises, while the belowground biomass for apical meristem removal demonstrates a cubic growth pattern with increasing pre‐grazing heights, stabilizing within the range of 24–35 cm (Figure 2b). Regardless of above‐ and belowground biomass, higher values are observed in cases where the apical meristem is conserved compared to its removal.

**TABLE 1:** Significance of the main effects and their interactions from the analysis of variance of above‐ and belowground biomass, herbage mass, herbage nutritive value, and herbage nutrient reserves in *Leymus chinensis* managed with different combinations of pre‐ and post‐grazing heights, in the entire growing season, during 2 years.

Parameters	Pre‐grazing height		Post‐grazing height		Year		Pre‐ × post‐grazing height		Pre‐grazing height × year		Post‐grazing height × year		Pre‐ ×post‐grazing height × year	
	*F*‐value	*p*‐Value	*F*‐value	*p*‐Value	*F*‐value	*p*‐Value	*F*‐value	*p*‐Value	*F*‐value	*p*‐Value	*F*‐value	*p*‐Value	*F*‐value	*p*‐Value
Aboveground biomass (g/m^2^)	43.470	<.001	12.347	.001	65.820	<.001	2.295	.082	5.339	.002	0.898	.351	1.427	.249
Belowground biomass (g/m^2^)	28.048	<.001	12.488	<.001	/	/	1.713	.198	/	/	/	/	/	/
CP content (%)	193.383	<.001	61.603	<.001	150.740	<.001	5.943	.001	79.864	<.001	6.042	.020	14.735	<.001
NDF content (%)	110.789	<.001	47.513	<.001	75.115	<.001	0.673	.616	48.234	<.001	3.869	.058	5.566	.002
ADF content (%)	93.440	<.001	1.198	.282	69.621	<.001	3.632	.015	54.475	<.001	29.601	<.001	27.630	<.001
ADL content (%)	174.783	<.001	36.137	<.001	8.714	.004	7.527	<.001	2.979	.024	0.001	.975	1.278	.285
Cumulative CP yield (g/m^2^)	73.355	<.001	17.109	<.001	490.384	<.001	0.873	.490	76.051	<.001	8.628	.006	4.059	.008
Cumulative NDF yield (g/m^2^)	60.094	<.001	7.845	.009	29.956	<.001	1.586	.204	3.643	.016	0.045	.834	2.847	.041
Cumulative ADF yield (g/m^2^)	80.975	<.001	6.336	.017	19.575	<.001	2.127	.102	4.988	.003	0.012	.914	4.939	.004
Cumulative ADL yield (g/m^2^)	180.812	<.001	0.037	.848	7.713	.007	2.434	0.057	1.234	.306	0.020	.887	1.531	.205
Soluble sugar content in stem (mg/g)	203.400	<.001	18.549	<.001	18.279	<.001	1.606	.198	10.333	<.001	0.046	.831	3.243	.025
Starch content in stem (mg/g)	355.179	<.001	4.546	.036	1.852	.179	1.268	.290	2.190	.081	0.671	.416	3.174	.020
Soluble sugar accumulation in stem (g/tiller)	137.878	<.001	14.463	.001	32.461	<.001	1.325	.297	7.074	<.001	1.620	.210	0.539	.708
Starch accumulation in stem (g/tiller)	121.535	<.001	47.764	<.001	47.673	<.001	3.180	.016	7.052	<.001	5.476	.022	4.594	.002
Soluble sugar content in leaf (mg/g)	369.192	<.001	10.215	.003	50.766	<.001	3.635	.014	4.148	.007	3.446	.072	0.620	.652
Starch content in leaf (mg/g)	347.577	<.001	9.626	.003	34.783	<.001	2.872	.028	6.239	<.001	0.193	.662	1.500	.213
Soluble sugar accumulation in leaf (g/tiller)	51.053	<.001	39.407	<.001	0.139	.710	3.183	.024	0.694	.600	3.235	.079	1.897	.127
Starch accumulation in leaf (g/tiller)	41.971	<.001	26.778	<.001	1.305	.259	2.889	.031	0.661	.622	6.194	.016	0.477	.752

### 3.2 Herbage mass and nutritive value of *Leymus chinensis*

**FIGURE 3:**
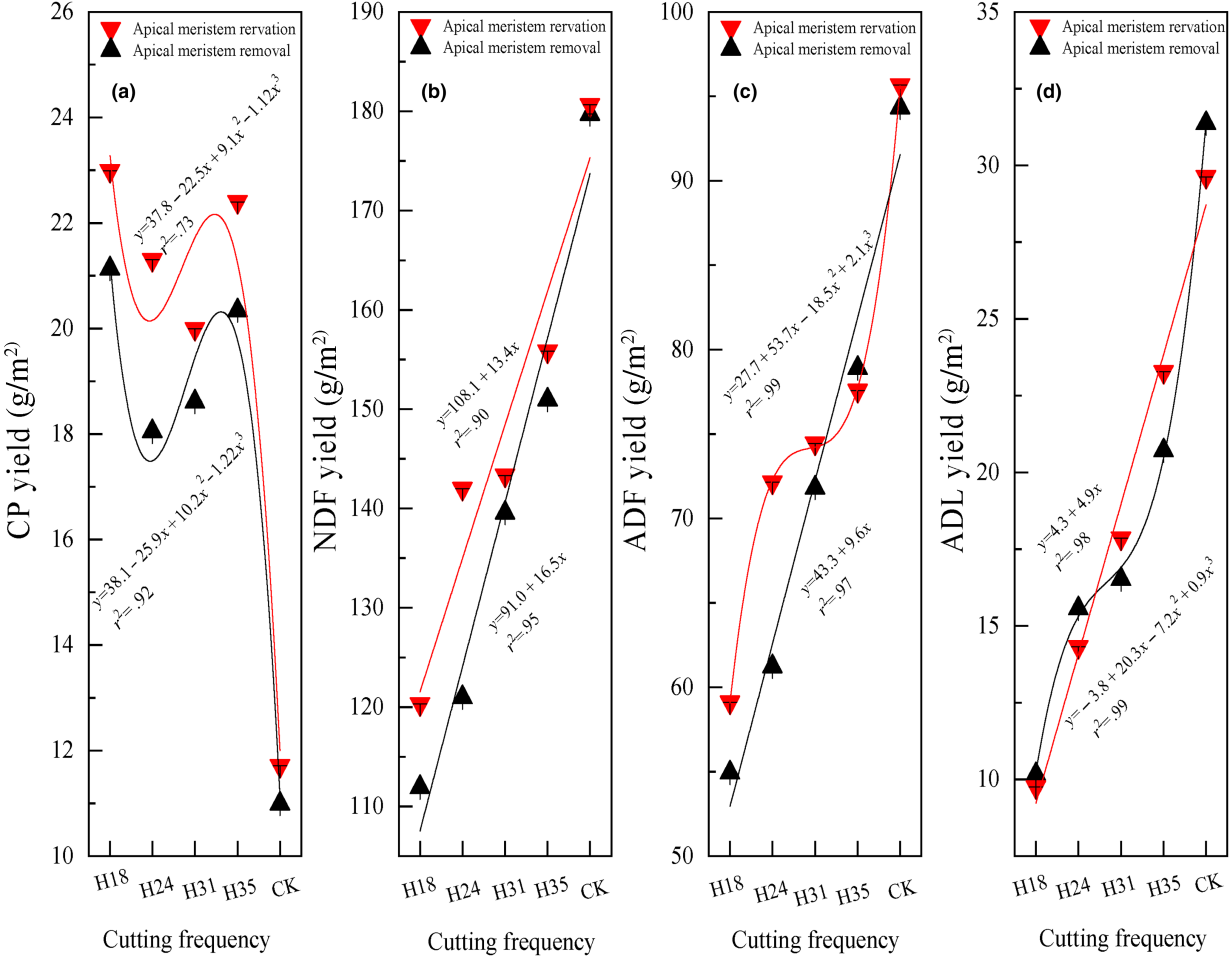
The crude protein (CP), neutral detergent fiber (NDF), acid detergent fiber (ADF), and acid detergent lignin (ADL) yields of *Leymus chinensis* in response to different pre‐grazing heights under two post‐grazing heights (apical meristem reservation and removal). The presented data in the figure represent the average values for each combination of pre‐ and post‐grazing height, considering all cutting frequencies within each year, and further averaged across 2 years. *Note*: H18 represents a pre‐grazing height of 18 cm; H24 represents a pre‐grazing height of 24 cm; H31 represents a pre‐grazing height of 31 cm; H35 represents a pre‐grazing height of 35 cm; CK represents no one cutting in mid‐August as common practice; apical meristem reservation denotes the post‐grazing height reserved for the apical meristem; and apical meristem removal denotes the post‐grazing height removed for the apical meristem.

The pre‐grazing height, post‐grazing height, and their interaction significantly impact the CP yield of *L*. *chinensis* (Table 1). Regardless of whether the apical meristem of *L*. *chinensis* is preserved or removed, the pre‐grazing height has a cubic effect on CP yield, with the highest CP yield observed at a pre‐grazing height of 18 cm and the lowest CP yield in CK (Figure 3a). The pre‐grazing height, post‐grazing height, and their interaction also have a significant influence on NDF yield and ADF yield. As the pre‐grazing height increases, there is a linear increase in NDF yield for both apical meristem removal and preservation (Figure 3b). However, for apical meristem reservation, there is a cubic increase in ADF yield while for apical meristem removal, there is a linear increase as the pre‐grazing height increases (Figure 3c). The pre‐grazing height and the interaction between pre‐ and post‐grazing height significantly influence ADL yield (Table 1). The variation in ADL yield exhibits an inverse relationship with ADF yield, as the ADL yield in apical meristem reservation increases linearly while the ADL yield in apical meristem removal increases cubically with increasing pre‐grazing height (Figure 3d). Overall, the CP, NDF, ADF, and ADL yields in apical meristem conservation at different pre‐grazing heights surpass those in apical meristem removal.

**FIGURE 4:**
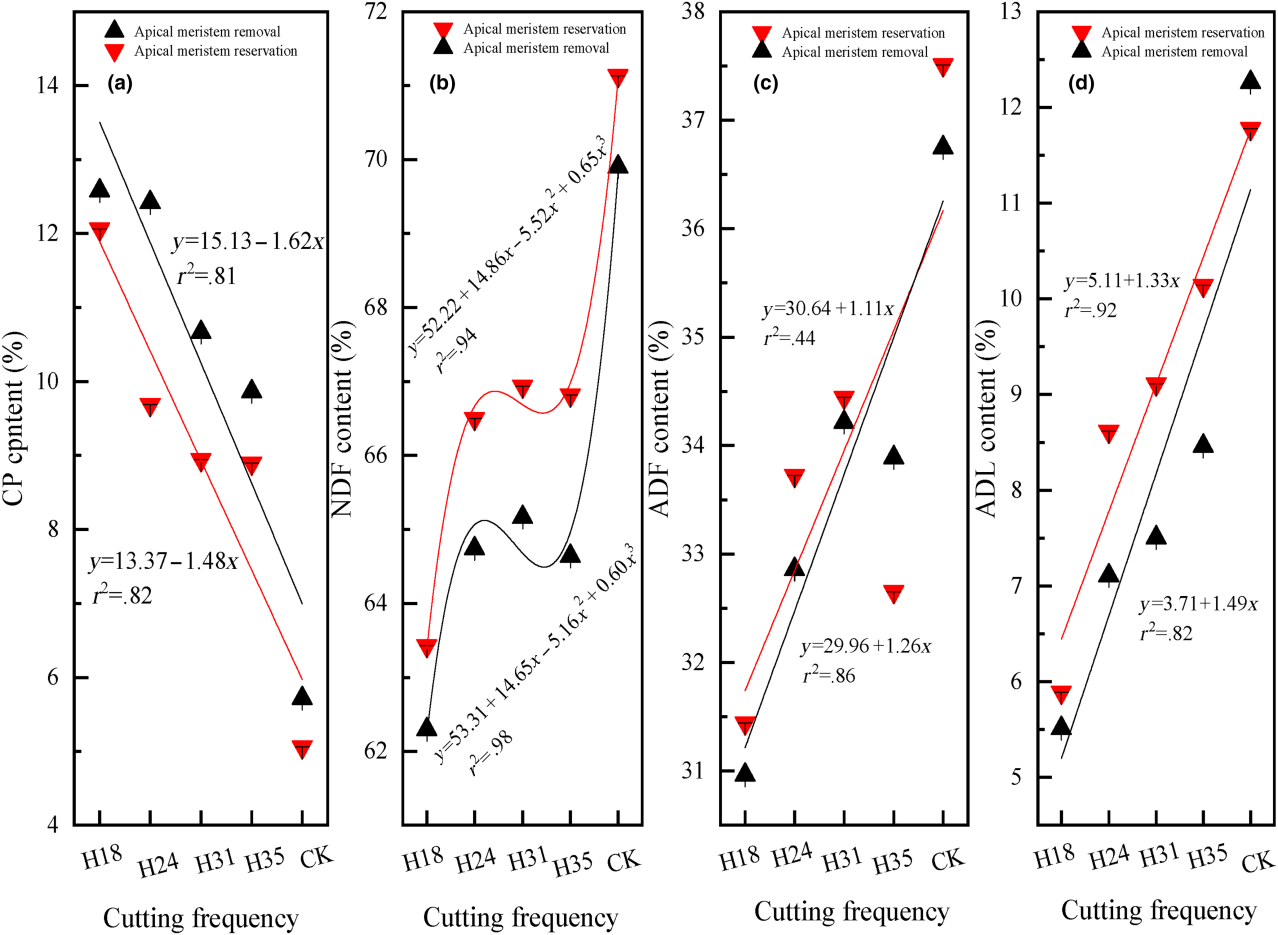
The crude protein (CP), neutral detergent fiber (NDF), acid detergent fiber (ADF), and acid detergent lignin (ADL) contents of *Leymus chinensis* in response to different pre‐grazing heights under two post‐grazing heights (apical meristem reservation and removal). The presented data in the figure represent the average values for each combination of pre‐ and post‐grazing height, considering all cutting frequencies within each year, and further averaged across 2 years. *Note*: H18 represents a pre‐grazing height of 18 cm; H24 represents a pre‐grazing height of 24 cm; H31 represents a pre‐grazing height of 31 cm; H35 represents a pre‐grazing height of 35 cm; CK represents no one cutting in mid‐August as common practice; apical meristem reservation denotes the post‐grazing height reserved for the apical meristem; and apical meristem removal denotes the post‐grazing height removed for the apical meristem.

The pre‐grazing height, post‐grazing height, and their interaction significantly influence the contents of CP, NDF, and ADL. Meanwhile, only the pre‐grazing height and the interaction between pre‐ and post‐grazing height have a significant effect on ADF content (Table 1). As the pre‐grazing height increases, there is a linear decrease in CP content for both apical meristem reservation and removal treatments while ADF and ADL contents show a linear increase for both treatments (Figure 4a,c,d). It is worth noting that regardless of apical meristem reservation or removal treatment, NDF content shows a cubic increase with an increasing pre‐grazing height (Figure 4b). Moreover, at different pre‐grazing heights, the CP content in apical meristem removal treatment is noticeably higher than that in apical meristem preservation treatment.

### 3.3 Plant soluble sugar and starch content and accumulation of *Leymus chinensis*

**FIGURE 5:**
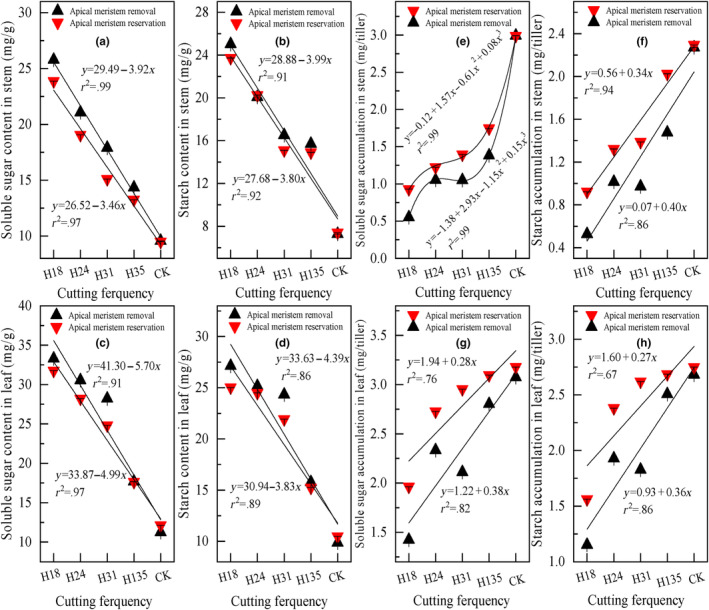
The soluble sugar contents and accumulations in stems (a, e) and leaves (c, g), as well as starch contents and accumulations in stems (b, f) and leaves (d, h) of *Leymus chinensis* in response to different pre‐grazing heights under two post‐grazing heights (apical meristem reservation and removal). The presented data in the figure represent the average values for each combination of pre‐ and post‐grazing height, considering all cutting frequencies within each year, and further averaged across 2 years. *Note*: H18 represents a pre‐grazing height of 18 cm; H24 represents a pre‐grazing height of 24 cm; H31 represents a pre‐grazing height of 31 cm; H35 represents a pre‐grazing height of 35 cm; CK represents no one cutting in mid‐August as common practice; apical meristem reservation denotes the post‐grazing height reserved for the apical meristem; apical meristem removal denotes the post‐grazing height removed for the apical meristem.

The pre‐grazing height and post‐grazing height both exert a significant influence on the soluble sugar and starch contents as well as their accumulations in stems and leaves. Additionally, the interaction between pre‐ and post‐grazing heights significantly affects all soluble sugar and starch contents and accumulations in stems and leaves, with the exception of starch content in stems and starch accumulation in leaves (Table 1). Generally, regardless of the preservation or removal of the apical meristem (Figure 5a–d), there is a decreasing trend in soluble sugar and starch contents in both stems and leaves with increasing pre‐grazing height. Conversely, there is an increasing trend in soluble sugar and starch accumulations in both stems and leaves with increasing pre‐grazing height, irrespective of the apical meristem reservation or removal (Figure 5e–h). Furthermore, as the pre‐grazing height increases, the soluble sugar and starch contents between apical meristem preservation and removal are hardly comparable. However, it is evident that the soluble sugar and starch accumulations in stems and leaves with apical meristem preservation are significantly higher than those with apical meristem removal.

## 4 DISCUSSION

Defoliation is commonly employed as a means to simulate grazing in experimental studies, with a typical stubble height is 5 cm (Song et al., 2018); however, this fails to accurately replicate the actual variability in grazing stubble height due to selective feeding by animals and fluctuations in plant height (Szymczak et al., 2020). The apical meristem of *L*. *chinensis* is located at the top of the stem and the apical meristem height progressively increases as the pre‐grazing height increases (Liu et al., 2019; Zhang et al., 2011). The relationship between pre‐and post‐grazing heights determines the reserve or removal of apical meristems, thereby affecting herbage yield and nutritive value, as well as soluble sugar and starch reserves of *L*. *chinensis*.

To preserve the apical meristem of *L*. *chinensis*, the post‐grazing should always maintain higher than its apical meristem height. Increasing the height of stubble leads to a decrease in the proportion of harvested aboveground biomass of *L*. *chinensis* to total biomass, resulting in a reduction in harvested forage yield (Yang et al., 2020). However, the results of this experiment showed that the cumulative herbage yield of *L*. *chinensis* was higher under preservation treatments of the apical meristem compared to removal treatments of the apical meristem. This indicated that the regeneration efficiency of *L*. *chinensis* was higher when the apical meristem was preserved, which makes up for the loss of cumulative of *L*. *chinensis* caused by the high stubble height. On the one hand, the preservation of the apical meristem of *L*. *chinensis* provides a material basis for rapid regeneration and can begin to rebuild the lost photosynthetic tissues within 2–6 days after defoliation. On the other hand, the presence of stems, facilitates the immediate mobilization of carbohydrates stored in them to replenish the photosynthetic apparatus, thereby enhancing growth rate (Oesterheld & McNaughton, 1988; Bihmidine et al., 2013; Silva et al., 2017). On the contrary, when the apical meristem of *L*. *chinensis* was removed, the *L*. *chinensis* died, and it took at least 2 weeks to reconstruct photosynthetic tissue by stimulating the stem of buds, which could consume a large amount of nutrients stored in the belowground of *L*. *chinensis* (Morvan‐Bertrand et al., 1999; Song et al., 2020).

Plant miniaturization is a growth strategy employed to mitigate defoliation stress, which in turn influences biomass allocation and nutrient distribution among organs due to allometric relationships (Li, Hou, et al., 2016; Li, Liu, et al., 2016; Liu et al., 2018). This growth strategy facilitates the mobilization of soluble sugar and starch reserved within plants, thereby accelerating the plant growth rate, and ultimately enhancing their nutritional value (Benot et al., 2019; Brink et al., 2013; Zhang et al., 2022). Reduced plant size implies higher defoliation intensity, resulting in faster growth rates and consequently greater nutritional value but lower yields (Zhang, He, et al., 2020). Therefore, in this experiment, the nutritional value (CP, NDF, ADF, and ADL) of *L*. *chinensis* was significantly enhanced under removal treatments of the apical meristem compared to preservation treatments; however, there was an opposite trend observed for cumulative DM yield. Melak et al. (2019) investigated the nutritive value of four herbaceous grass species (*Cynodon dactylon*, *Sporobolus pyramidalis*, *Setaria verticillata*, and *Digitaria ternata*) under varying levels of defoliation. The findings also revealed that lower grazing intensity led to higher contents of NDF, ADF, and ADL in the forage compared to higher grazing intensity. de Almeida et al. (2023) discovered that the herbage under continuous grazing exhibited a higher nutritive value compared to rational grazing, indicating a greater grassland renewal potential for continuous grazing. Moreover, after the removal of the apical meristem *L*. *chinensis*, the rhizome buds located belowground tend to generate new tillers, whereas if the apical meristem is preserved, *L*. *chinensis* tends to continue growing and consequently exhibits low rates of tiller renewal (Liu et al., 2019). This means the *L*. *chinensis* under reservation treatments of the apical meristem tends to retain older tillers, which could explain the lowest nutritional value obtained from these treatments.

The herbage yield and nutritive value demonstrated an inverse correlation with defoliation intensity, thus necessitating trade‐offs in managing productivity and nutritive value to optimize the sustainability of *L*. *chinensis* grassland (Bekewe et al., 2020). Herbage mass can serve as an integrated indicator encompassing herbage yield and nutritive value factors, yet its response to defoliation intensity may exhibit more intricate variations compared to herbage yield. Previous studies have indicated that the reduction in plant yield can be attributed to the key mechanism of plant miniaturization induced by increasing defoliation intensity (Zhao et al., 2022). In the present experiment, there was a consistent decrease in cumulative herbage yield with increasing defoliation intensity (from 18 cm pre‐grazing height to 35 cm pre‐grazing height) in both years. However, regardless of the post‐grazing plant height (2 or 6 cm) in 2020, the highest cumulative CP yield was observed at 18 cm pre‐grazing height, representing the maximum defoliation intensity. Nevertheless, a sharp decline in CP yield was observed specifically in the treatments of 18 cm pre‐grazing height with a 2 cm or 6 cm post‐grazing height in 2021. This is because the variation in CP content of 18 cm pre‐grazing height was primarily responsible for the variability in cumulative CP yield of *L*. *chinensis*. Although a pre‐grazing height of 18 cm resulted in the highest number of frequent cuttings, it did not provide any advantages in terms of cumulative herbage yield, as the highest values were consistently observed at both the control treatment and the treatment with a pre‐grazing height of 35 cm, regardless of the post‐grazing height. Furthermore, the combination of the post‐grazing height of 17 cm and pre‐grazing height of 35 cm maintained a stable CP yield across multiple years under all defoliation treatments, making it an optimal defoliation regime for managing *L*. *chinensis* grassland.

Currently, the traditional and practical practice of annual autumn hay making is prevalent in arid and semi‐arid grasslands. However, due to the low nutritive value of harvested forage, the conversion efficiency of forage is restricted and cannot meet the nutritional requirements of animals. Defoliation is a viable means of enhancing forage nutritive value. However, a multitude of experimental findings indicate that defoliation regimes vary across different regions due to diverse climatic conditions and herbage types (Baoyin et al., 2014; Holechek & Pieper, 2011). Besides, continuous defoliation may have a detrimental effect on cumulative forage yield over time and became more pronounced in the 2nd year (Zhang, Li, et al., 2020). Additionally, the belowground biomass of *L*. *chinensis* serves as a crucial indicator for predicting the sustainability of *L*. *chinensis* grassland (Li, Hou, et al., 2016; Li, Liu, et al., 2016). Although the findings of the present study showed that there was no significant decrease in belowground *L*. *chinensis* in the combination of the post‐grazing height of 17 cm and pre‐grazing height of 35 cm compared to control treatment in both years, it is necessary to allow for a recovery period after continuous defoliation. The objective of this initiative is to proactively mitigate the risk of grasslands deteriorating to a critical level, considering that the restoration procedure for degraded grassland entails a prolonged long‐term (Vecchio et al., 2019). Furthermore, in order to achieve sustainable grassland management, future studies should focus on determining the optimal combination of years for continuous defoliation and restoration in mowed grasslands.

## 5 CONCLUSION

Preserving and removing the apical meristem of *L*. *chinensis* exert a significant influence on its CP and fiber yield, as well as the soluble sugar and starch reserves within plants. Increasing defoliation intensity results in reduced plant size, thereby enhancing nutritional value while compromising yields. Although defoliation at a pre‐grazing height of 18 cm resulted in the highest number of frequent cuttings, it did not confer any advantages in terms of cumulative herbage yield as the highest values were observed at control treatment and the treatment of 35 cm pre‐grazing height, regardless of whether the apical meristem of *L*. *chinensis* was preserved or removed in both years. Furthermore, the combination of the post‐grazing height of 17 cm and pre‐grazing height of 35 cm demonstrated consistent CP yield across 2 consecutive years under all defoliation treatments. Further experimental investigations are imperative to elucidate long‐term grassland management and establish optimal grazing regimes in terms of consecutive years of defoliation and restoration.

## AUTHOR CONTRIBUTIONS

**Chengzhen Zhao:** Data curation (equal); methodology (lead); software (equal); visualization (lead); writing – original draft (lead). **Xiao Chang:** Data curation (equal); software (equal); writing – review and editing (equal). **Qiang Li:** Methodology (equal); software (equal); writing – review and editing (equal). **Rongzhen Zhong:** Funding acquisition (equal); writing – review and editing (equal). **Daowei Zhou:** Funding acquisition (equal); methodology (equal); project administration (equal); writing – review and editing (equal).

## FUNDING INFORMATION

This work was financially supported by the National Key Research and Development Program (2022YFD1500505), the Natural Science Foundation of Jilin Province (General Project of Free Exploration) (YDZJ202301ZYTS350), the Strategic Priority Research Program of the Chinese Academy of Sciences (XDA26040305), and the Shennong Talent Plan of the Ministry of Agriculture and Rural Affairs (SNYCQN138‐2022).

## CONFLICT OF INTEREST STATEMENT

The authors declare no conflict of interest.

### OPEN RESEARCH BADGES

The article has earned an Open Data badge for providing publicly accessible digitally‐shareable data necessary to replicate the reported results. The data is available at: https://doi.org/10.6084/m9.figshare.23515884.v3. The supporting information can be found in the Appendix S1.

## Supporting information


Appendix S1.

## Data Availability

The complete manuscript data can be accessed at https://doi.org/10.6084/m9.figshare.23515884.v3.
