# Supplementary material for: Effects of different combinations of pre‐ and post‐grazing heights on herbage mass and nutrient reserves of Leymus chinensis in Northeast China
Source: Ecol Evol. 2024 May 6;14(5):e11336. doi: 10.1002/ece3.11336 (PMC11070838; doi:10.1002/ece3.11336)
Supplement: Supplementary file 1 — Appendix S1. [file ECE3-14-e11336-s001.docx]

**Determination of pre-grazing heights and their corresponding apical meristem heights**

From May 1, 2020 to June 24, 2020, in a grassland of 0.3 ha, pre-grazing plant height and apical meristem height of *L. chinensis* were measured at four-day intervals. On each sampling date, a total of 100 randomly selected complete and consistent individual *L. chinensis* plants were mowed at ground level and immediately transferred to the laboratory. For each individual plant, the initial measurement was taken for plant height before carefully peeling its fully unfolded and partially unfolded leaves until apical meristem was exposed; subsequently, the apical meristem height was measured. The results indicated that pre-grazing plant heights of *L. chinensis* on May 15, May 27, June 3, June 10 were measured at 18 cm, 24 cm, 31 cm, and 35 cm, respectively. Correspondingly, the apical meristem heights accounted for approximately 9.4% (1.7 cm), 24.2% (5.8 cm), 30.6% (9.5 cm), and 39.1% (13.7 cm) of the pre-grazing plant heights of 18 cm, 24 cm, 31 cm, and 35 cm, respectively. The observed variation gradient in these pre-grazing plant heights effectively reflected the progression of plant development as further growth in height was not significantly evident.

**Determination of the post-grazing heights corresponding to each pre-grazing height**

A low grazing intensity was conducted to determine the post-grazing height/defoliation height required for reserving the apical meristem at different pre-grazing plant heights, namely 18 cm, 24 cm, 31 cm, and 35 cm. The paddock was 10 m × 10 m, with 4 paddocks for each pre-grazing plant height, totaling 16 paddocks. Three healthy adult Ujumuqin ewes were rotated across four paddocks (one hour per paddock) from 7 a.m. to 11 a.m., at each pre-grazing plant height. Subsequently, the post-grazing plant heights of L chinensis within each paddock (25 measurements per paddock) and a total of 100 post-grazing plant heights across all four paddocks were recorded. The results indicated that the post-heights accounted for approximately 33.3% (6 cm), 41.7% (10 cm), 43.45% (13.5 cm), and 48.6% (17 cm) of the pre-grazing plant heights of 18 cm, 24 cm, 31 cm, and 35 cm, respectively. Consequently, these post-grazing heights would be capable of reserving the intactness of apical meristems. On the other hand, the post-grazing height of 2 cm was appropriated to remove the remove apical meristem of *L. chinensis*, regardless pre-grazing heights (i.e., 18 cm, 24 cm, 31 cm, and 35 cm). Therefore, post-grazing height accounted for approximately 11.1%, 8.3%, 6.5% and 5.7% of the pre-grazing plant heights of 18 cm, 24 cm, 31cm and 35 cm, respectively resulting in the removal of apical meristem from all pre-grazing plant heights.

Supplementary TABLE 1 Plant mowing and sampling dates in 2020 and 2021.

| Treatments | | 2020 |  |  |  |  |  | 2021 |  |  |  |  |  |
| --- | --- | --- | --- | --- | --- | --- | --- | --- | --- | --- | --- | --- | --- |
|  |  | Sampling and mowing dates | | |  |  |  | Sampling and mowing dates | | |  |  |  |
| RM treatments | |  |  |  |  |  |  |  |  |  |  |  |  |
| RM/CK |  |  |  |  | 8/15 (M, I) |  |  |  |  |  | 8/15 (M, I) |  | R |
| RM/H18 |  | 5/15 (M, I) | 6/4 (M, I) | 6/30 (M, I) | 8/19 (M, I) | 9/30 (M) |  | 6/12 (M, I) | 7/13 (M, I) |  |  |  | 9/30 (M, R) |
| RM/H24 |  | 5/28 (M, I) | 6/30 (M, I) | 8/19 (M, I) |  | 9/30 (M) |  | 6/12 (M, I) | 8/21 (M, I) |  |  |  | 9/30 (M, R) |
| RM/H31 |  | 6/4 (M, I) | 7/15 (M, I) |  |  | 9/30 (M) |  | 6/28 (M, I) | 8/21 (M, I) |  |  |  | 9/30 (M, R) |
| RM/H35 |  | 6/11 (M, I) |  |  |  | 9/30 (M) |  | 7/11 (M, I) |  |  |  |  | 9/30 (M, R) |
| RS treatments | |  |  |  |  |  |  |  |  |  |  |  |  |
| RS/CK |  |  |  |  | 8/15 (M, I) |  |  |  |  |  | 8/15 (M, I) |  | R |
| RS/H18 |  | 5/15 (M, I) | 6/4 (M, I) | 6/27 (M, I) | 7/15(M, I) | 9/30 (M) |  | 6/1 (M, I) | 6/28 (M, I) | 8/8 (M, I) |  |  | 9/30 (M, R) |
| RS/H24 |  | 5/28 (M, I) | 6/27 (M, I) | 7/15 (M, I) |  | 9/30 (M) |  | 6/1 (M, I) | 7/13 (M, I) |  |  |  | 9/30 (M, R) |
| RS/H31 |  | 6/4 (M, I) | 6/27 (M, I) | 8/1 (M, I) |  | 9/30 (M) |  | 6/12 (M, I) | 8/8 (M, I) |  |  |  | 9/30 (M, R) |
| RS/H35 |  | 6/11 (M, I) | 6/30 (M, I) | 8/19 (M, I) |  | 9/30 (M) |  | 6/28 (M, I) | 8/8 (M, I) |  |  |  | 9/30 (M, R) |

*Note*. M, defoliation and sampling dates of aboveground of *Leymus chinensis*; I, sampling dates of individual tiller of *L. chinensis*; R, sampling dates of belowground. For RM treatments, RM/CK represents 6 cm defoliation height applied on mid-August; RM/H18 represents a combination of pre-grazing at 18 cm and post grazing height at 2 cm; RM/H24 represents a combination of pre-grazing at 24 cm and post grazing height at 2 cm; RM/H31 represents a combination of pre-grazing at 31 cm and post grazing height at 2 cm and RM/H35 represents a combination of pre-grazing at 35 cm and post grazing height at 2 cm; For RS treatments, RS/CK was equivalent with RM/CK; RS/H18 represents a combination of pre-grazing at 18 cm and post grazing height at 6 cm; RS/H24 represents a combination of pre-grazing at 24 cm and post grazing height at 10 cm; RS/H31 represents a combination of pre-grazing at 31 cm and post grazing height at 13.5 cm and RS/H35 represents a combination of pre-grazing at 35 cm and post grazing height at 17 cm.

Supplementary TABLE 2 Variations of pre-grazing height and apical meristem height of all treatments at each mowing date in 2020 and 2021

| Treatments | | 2020 | | | |  | 2021 | | |
| --- | --- | --- | --- | --- | --- | --- | --- | --- | --- |
| RM/H18 | Mowing date | 5/15 | 6/4 | 6/30 | 8/19 |  | 6/12 | 7/13 |  |
|  | Pre-grazing height (cm) | 18.2 | 18.8 | 18.6 | 17.7 |  | 19.4 | 18.1 |  |
|  | Apical meristem height (cm) | 1.7 | 2.3 | 2.6 | 4.1 |  | 2.7 | 2.0 |  |
| RM/H24 | Mowing date | 5/28 | 6/30 | 8/19 |  |  | 6/12 | 8/21 |  |
|  | Pre-grazing height (cm) | 23.8 | 23.6 | 23.7 |  |  | 23.6 | 24.1 |  |
|  | Apical meristem height (cm) | 5.8 | 5.1 | 4.9 |  |  | 5.2 | 4.9 |  |
| RM/H31 | Mowing date | 6/4 | 7/15 |  |  |  | 6/28 | 8/21 |  |
|  | Pre-grazing height (cm) | 31.0 | 31.4 |  |  |  | 30.8 | 30.6 |  |
|  | Apical meristem height (cm) | 9.5 | 8.1 |  |  |  | 9.6 | 7.9 |  |
| RM/H35 | Mowing date | 6/11 |  |  |  |  | 7/11 |  |  |
|  | Pre-grazing height (cm) | 35.8 |  |  |  |  | 34.5 |  |  |
|  | Apical meristem height (cm) | 13.7 |  |  |  |  | 13.0 |  |  |
| RS/H18 | Mowing date | 5/15 | 6/4 | 6/27 | 7/15 |  | 6/1 | 6/28 | 8/8 |
|  | Pre-grazing height (cm) | 18.2 | 19.7 | 19.4 | 17.9 |  | 18.2 | 19.3 | 18.7 |
|  | Apical meristem height (cm) | 1.7 | 2.7 | 4.0 | 4.5 |  | 2.2 | 3.8 | 4.7 |
| RS/H24 | Mowing date | 5/28 | 6/27 | 7/15 |  |  | 6/1 | 7/13 |  |
|  | Pre-grazing height (cm) | 23.8 | 24.9 | 24.8 |  |  | 23.2 | 23.2 |  |
|  | Apical meristem height (cm) | 5.8 | 6.9 | 8.1 |  |  | 5.4 | 8.0 |  |
| RS/H31 | Mowing date | 6/4 | 6/27 | 8/1 |  |  | 6/12 | 8/8 |  |
|  | Pre-grazing height (cm) | 31.0 | 31.4 | 30.9 |  |  | 30.8 | 30.9 |  |
|  | Apical meristem height (cm) | 9.5 | 10.5 | 13.5 |  |  | 10.3 | 11.0 |  |
| RS/H35 | Mowing date | 6/11 | 6/30 | 8/19 |  |  | 6/28 | 8/18 |  |
|  | Pre-grazing height (cm) | 35.8 | 35.7 | 35.0 |  |  | 33.6 | 35.5 |  |
|  | Apical meristem height (cm) | 13.7 | 15.0 | 16.8 |  |  | 13.4 | 15.5 |  |
